# Paradox of AI in Higher Education: Qualitative Inquiry Into AI Dependency Among Educators in Palestine

**DOI:** 10.2196/74947

**Published:** 2025-09-15

**Authors:** Anas Ali Alhur, Zuheir N Khlaif, Bilal Hamamra, Elham Hussein

**Affiliations:** 1 Imam Abdulrahman Bin Faisal University, Damam, Saudi Arabia; 2 Educational Sciences, An-Najah National University, Old Campus Street, Nablus, West Bank, P358, Palestinian Territory, 970 0592754908; 3 Education Language, An-Najah National University, Nablus, West Bank, Palestinian Territory; 4 Al Ain University, Al Ain, United Arab Emirates

**Keywords:** AI dependency, generative AI, procrastination, AI reliance, hybrid intelligence

## Abstract

**Background:**

Artificial intelligence (AI) is increasingly embedded in medical education, providing benefits in instructional design, content creation, and administrative efficiency. Tools like ChatGPT are reshaping training and teaching practices in digital health. However, concerns about faculty overreliance highlight risks to pedagogical autonomy, cognitive engagement, and ethics. Despite global interest, there is limited empirical research on AI dependency among medical educators, particularly in underrepresented regions like the Global South.

**Objective:**

This study focused on Palestine and aimed to (1) identify factors contributing to AI dependency among medical educators, (2) assess its impact on teaching autonomy, decision-making, and professional identity, and (3) propose strategies for sustainable and responsible AI integration in digital medical education.

**Methods:**

A qualitative research design was used, using semistructured interviews (n=22) and focus group discussions (n=24) involving 46 medical educators from nursing, pharmacy, medicine, optometry, and dental sciences. Thematic analysis, supported by NVivo (QSR International), was conducted on 15.5 hours of transcribed data. Participants varied in their frequency of AI use: 45.7% (21/46) used AI daily, 30.4% (14/46) weekly, and 15.2% (7/46) monthly.

**Results:**

In total, 5 major themes were identified as drivers of AI dependency: institutional workload (reported by >80% [37/46] of participants), low academic confidence (noted by 28/46, 60%), and perfectionism-related stress (23/46, 50%). The following 6 broad consequences of AI overreliance were identified: Skills Atrophy (reported by 89% [41/46]): educators reported reduced critical thinking, scientific writing, and decision-making abilities. Pedagogical erosion (35/46, 76%): decreased student interaction and reduced teaching innovation. Motivational decline (31/46, 67%): increased procrastination and reduced intrinsic motivation. Ethical risks (24/46, 52%): concerns about plagiarism and overuse of AI-generated content. Social fragmentation (22/46, 48%): diminished peer collaboration and mentorship. Creativity suppression (20/46, 43%): reliance on AI for content generation diluted instructional originality.

Strategies reported by participants to address these issues included establishing boundaries for AI use (n=41), fostering hybrid intelligence (n=37), and integrating AI literacy into teaching practices (n=39).

**Conclusions:**

While AI tools can enhance digital health instruction, unchecked reliance risks eroding essential clinician competencies. This study identifies cognitive, pedagogical, and ethical consequences of AI overuse in medical education and highlights the need for AI literacy, professional development, and ethical frameworks to ensure responsible and balanced integration.

## Introduction

### Background

The rapid incorporation of artificial intelligence in education (AIED) has significantly changed the educational landscape, providing unique chances for tailored teaching and learning experiences, instant feedback, and enhanced efficiency [1]. Consequently, institutions across the globe are increasingly using AI-powered tools, including generative models like ChatGPT, to customize the educational process, streamline administrative duties, and reduce faculty workload [2]. Despite important benefits, stakeholders have concerns regarding overdependence on AI, which is simply defined as students’ and instructors’ uncritical acceptance of AI-generated material [3]. In addition, overreliance on AIED hinders cognitive involvement, productive critical thinking and decision making, and long-term skill growth [4].

While literature on the topic of AIED is abundant, it primarily focuses on the gains of integrating AI in the educational setting [5]. Ethical, cognitive, and professional challenges related to AIED, on the other hand, are far less explored [6,7]. Furthermore, most of the available research on overreliance on AIED is investigated in relation to students, exploring how excessive dependence on AI can negatively impact their ability to think critically and independently and to perform self-directed learning [4,8]. Other student-related studies have linked AI reliance to psychological factors such as perfectionism, impulsivity, and the need for immediate cognitive relief, drawing comparisons to internet and social media dependence [9].

Empirical research investigating AI dependency among educators is still meager [7], and most of the existing studies explore how AI enhances educators’ productivity and decision-making [10]. Risks and challenges pertaining to AIED, on the other hand, are less explored. In addition, most research on AIED has been conducted in Western or Chinese contexts, with limited cross-cultural perspectives [11]. This study has three aims: to examine the causes of educators’ overreliance on AI, to assess its impact on teaching autonomy and pedagogical identity, and to identify challenges and strategies for responsible AI use. Focusing on Palestinian higher education, the research offers insights to guide policy, faculty development, and ensure that AI enhances—rather than replaces—educators’ professional expertise.

This study conceptualizes the “AI Paradox” in higher education, where AI’s benefits in streamlining academic work are offset by concerns over skill atrophy, ethics, and autonomy. It explores how faculty navigate these competing effects, highlighting the coexistence of innovation and emerging professional vulnerabilities.

### Research Questions

The research questions are as follows (1) What key factors contribute to AI dependency among educators in higher education? (2) How does AI dependency impact educators’ teaching autonomy, decision-making, and professional identity? (3) What strategies help educators maintain a balanced use of AI while maintaining pedagogical creativity and instructional effectiveness?

### Theoretical Foundation of the Study

**Figure 1.**
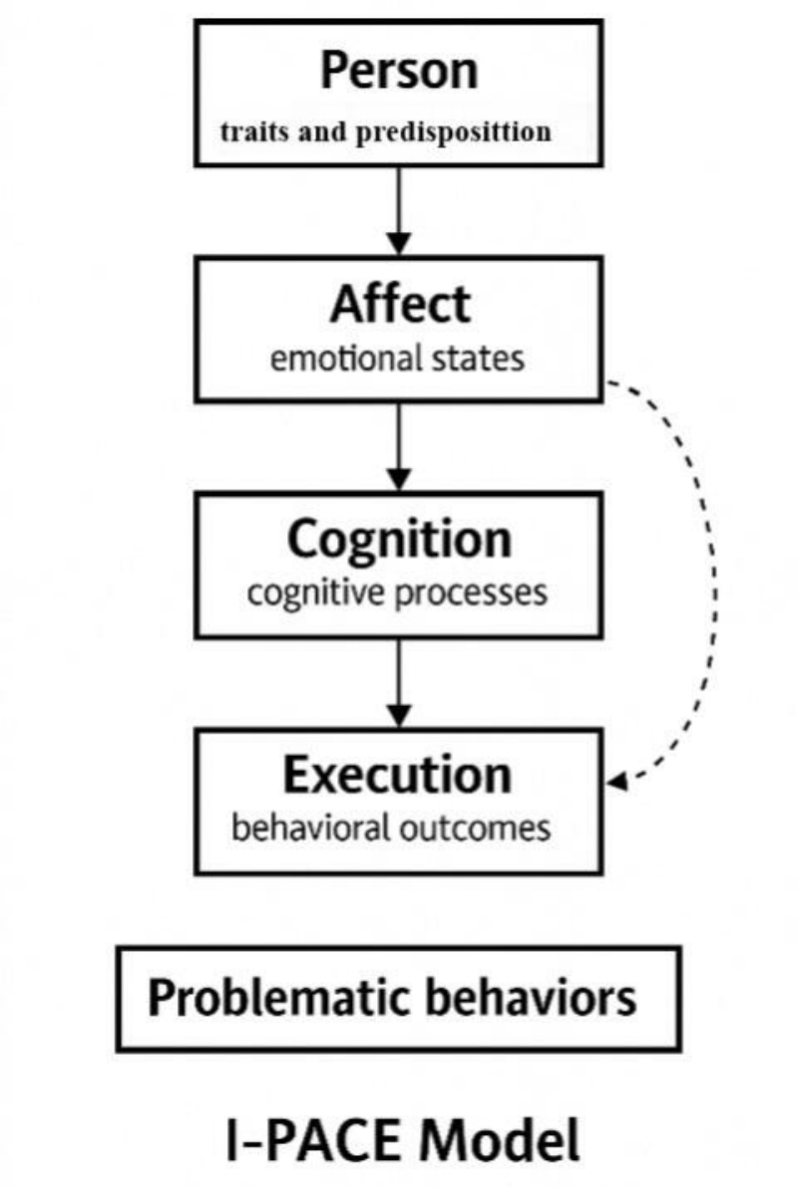
I-PACE Model. I-PACE: Interaction of Person-Affect-Cognition-Execution.

The Interaction of Person-Affect-Cognition-Execution (I-PACE Model; Figure 1), developed by [12], provides a comprehensive framework for understanding problematic technology use, including AI dependency. The I-PACE model explains how individual traits, emotional responses, cognitive processes, and behaviors interact to shape technology reliance. Its 4 components are: person-level predispositions (personality traits and cognitive styles), affect (emotional states like stress or anxiety), cognition (decision-making and impacts on autonomy), and execution (behavioral outcomes such as deskilling). This framework helps analyze how and why AI dependency develops among educators and its effects on autonomy and professional agency.

### Previous Studies Using I-PACE

Research using I-PACE has demonstrated how individual predispositions, emotional states, and cognitive processes interact to influence excessive reliance on digital tools. For instance, studies on internet and social media addiction have found that individuals with high neuroticism**,** impulsivity, or perfectionism are more likely to develop problematic usage patterns, often as a coping mechanism for stress [12]. Similarly, research on AI reliance in education suggests that educators and students with low self-efficacy in digital literacy or high performance expectations tend to offload cognitive tasks to AI, reducing engagement in critical thinking and problem-solving [8,13]. Studies show that institutional pressures and workload demands can drive educators to rely on AI for efficiency, often at the expense of pedagogical autonomy. Applying the I-PACE model, these studies highlight how occasional AI use can evolve into habitual dependence, shaped by psychological, cognitive, and behavioral factors.

Reliance on AI in education may lead to unhealthy dependence on AI tools that can reduce student autonomy, hinder skill acquisition, and elevate the risk of academic stress [8,14]. Although tactical application of AI is likely to improve teaching effectiveness and student engagement, students could still delegate crucial cognitive activities to technology rather than participating in reflective or creative thought [9,15,16]. When consistently depending on AI for writing tasks, research projects, or making decisions, students jeopardize their chances of developing critical thinking, creativity, and self-directed learning techniques [4,16]. While AI can serve as a valuable complement to teaching strategies, instructors must ensure that embracing technology does not impede human-centered exploration [17].

The psychological implications of reliance on AI are complex, involving personality characteristics, emotional control, and cognitive distortions [8]. According to [7] and [8], neuroticism, self-critical perfectionism, and impulsivity elevate the chances of excessive dependence on AI-driven tools. Neuroticism, marked by increased responses to stress, frequently leads individuals to pursue immediate technological solutions for anxiety or performance apprehensions [18]. Similarly, self-critical perfectionists might be drawn to the flawless results that AI is thought to produce, seeking to prevent errors and the unease linked to learning through trial and error [19]. Instead of developing study habits, impulsive learners might resort to AI whenever they face challenges, perpetuating a pattern of dependence on external sources. This AI dependency diminishes creativity and independent thought [8].

Scholars use the I-PACE model to explore how personal traits—like perfectionism and impulsivity—interact with emotional states and cognitive functions and sometimes result in maladaptive technology use [8,12]. This model illustrates how some individuals deal with academic pressure by consistently looking for digital shortcuts. Depending on digital shortcuts compromises an individual’s need for autonomy, competence, and relatedness, which, according to the Basic Psychological Needs theory, are crucial for one’s well-being and motivation [20]. If AI reliably addresses unmet needs—like providing quick responses that protect learners from the unease of ambiguity—an excessive dependence might develop, reducing the intrinsic motivation essential for authentic learning and developmental progress [8,13]. Simultaneously, determining which aspects of AI design—such as user interface, adaptive feedback, or customization features—enhance or reduce dependency could assist developers in creating more ethically responsible platforms [21].

Adverse academic emotions—like anxiety and frustration—can greatly heighten reliance on AI, hindering students’ motivation and their capacity to manage their own learning [22]. As unaddressed psychological needs build up, students might seek emotional support or affirmation from AI [8,23]. Performance expectations further amplify this dependence: when students think AI significantly enhances their grades or educational results, they might overrate the technology’s necessity [24]. This corresponds with extensive research on performance expectancy, an element demonstrated to be essential in the adoption and ongoing usage of new technologies [25]. Eventually, this persistent trend of pursuing immediate solutions or emotional comforts causes learners to neglect crucial cognitive activities like integrating information or contemplating mistakes [8].

## Methods

### Overview

This study used a qualitative research approach to explore the factors influencing AI dependency and the consequences of AI dependency in medical education. A mixed approach of semistructured interviews and focus group sessions was used to gain insight into the experiences and perspectives of faculty members and to explore how using AI tools in their practice affects them as educators [26].

To ensure participant anonymity while distinguishing between data sources, a consistent coding system was used. “E” refers to individual educators who participated in semistructured interviews, followed by a number identifying the participant (eg, E5=Educator 5). **“**EFG” refers to participants in focus group discussions (eg, EFG14). This system enabled pattern recognition across data types while maintaining confidentiality.

### Semistructured Interviews

Semistructured interviews were conducted with 22 faculty members to explore factors driving AI dependency and its consequences in medical education [27]. Participants were purposively sampled from multiple universities and represented diverse disciplines (eg, nursing, pharmacy, optometry, medicine, and dental sciences). Variation in roles, teaching experience, and frequency of AI use was considered to ensure maximum variation and minimize overlap in perspectives due to shared institutional or disciplinary contexts. The interview protocol (Multimedia Appendix 1) was informed by the study’s theoretical framework. Each interview lasted 25‐35 minutes and was audio-recorded with consent. The interview guide (Multimedia Appendix 1) was informed by the I-PACE framework and developed to explore individual, cognitive, and emotional dimensions of AI use. This guided the formulation of probes around motivation, attitudes, and usage patterns.

### Focus Group Sessions

To complement the interviews, 4 focus group sessions (n=24) were held with educators not involved in the interviews. This method enabled reflection and interaction, encouraging participants to build on shared experiences [28-31]. Purposive sampling ensured diversity across institutions, disciplines, and levels of AI engagement. All participants held teaching, research, or administrative roles. Focus group prompts (Multimedia Appendix 2) were generated from preliminary interview analysis to deepen insight into shared institutional experiences. Sessions were audio-recorded and lasted approximately 1 hour. In total, the study involved 46 participants.

### Data Collection

In total, 22 semistructured interviews (25-35 min each) were audio-recorded with consent. Questions explored participants’ use of generative AI in teaching, research, and administration, including frequency, delegated tasks, and changing reliance. Follow-ups addressed motivations, benefits, and challenges in maintaining professional autonomy alongside AI integration.

Although the average duration of these interviews may appear relatively short for qualitative research, this was a deliberate methodological decision based on both logistical and conceptual considerations. Participants were full-time faculty members in medical and health sciences disciplines with heavy teaching, research, and clinical responsibilities. To respect their limited availability and ensure meaningful engagement, a focused and well-structured interview protocol was designed, aligned with the I-PACE model. This protocol was pilot-tested and refined to elicit conceptually rich responses within a concise time frame.

Despite the shorter duration, the interviews yielded dense, thematically robust narratives. To enrich data depth and validate emerging themes, 4 focus group discussions—each approximately 1 hour—were also conducted. These provided opportunities for reflection, triangulation, and deeper elaboration of core issues. Importantly, thematic saturation was achieved after 18 interviews, with subsequent interviews and focus groups confirming the stability of the thematic framework. Thus, while longer interviews might have allowed for further elaboration, the approach adopted ensured a balance between feasibility and qualitative rigor, without compromising the credibility or conceptual depth of the data.

To supplement these insights, four 1-hour focus groups (2 in person and 2 online) with 24 participants were held, facilitated by 2 researchers using prompts from interview analysis. Discussions explored AI usage patterns, reliance, and impacts on teaching and decision-making. All sessions were audio-recorded, providing broader perspectives that complemented the interview findings.

To ensure conceptual depth and data adequacy, recruitment and data collection continued until thematic saturation was achieved. Saturation was defined as the point at which no new themes or insights emerged during ongoing data analysis. This was observed after conducting 18 interviews, with subsequent interviews and focus group discussions confirming the stability and completeness of the thematic framework.

In this study, AI dependency is defined as overreliance on generative AI tools for academic tasks, leading to reduced cognitive engagement, professional autonomy, or pedagogical creativity. This contrasts with effective AI integration, which involves strategic, ethical use that maintains human oversight. Semistructured interviews probed these distinctions, exploring both the benefits and drawbacks of AI use and assessing impacts on teaching autonomy, motivation, and professional identity.

### Data Analysis

An inductive thematic analysis was conducted following Braun and Clarke’s 6-phase framework (2006) to analyze data. The dataset comprised 12.25 hours of interview recordings and 3.25 hours of focus group sessions. All recordings were transcribed verbatim and verified through participant member checking to ensure accuracy.

Coding was performed using NVivo, with initial codes iteratively refined into subthemes and themes aligned with study objectives and relevant literature. Data collection and analysis were concurrent, and thematic saturation was reached after the 18th interview, confirmed by later focus groups.

To strengthen credibility, the study used methodological triangulation with interviews, focus groups, and artifacts such as anonymized AI-generated lesson plans and teaching notes. Analyzing these artifacts alongside participant reports provided contextual depth and validated emerging themes. Preserving cultural and linguistic nuances, coding and theme development were conducted in Arabic by bilingual researchers, with themes collaboratively translated into English. Backward translation ensured accuracy and contextual depth, while team discussions resolved discrepancies, maintaining conceptual equivalence and data integrity.

Participant responses were categorized using standardized terms: “most” (35/46, >75%), “many” (50% [23/46,]‐75% [35/46]), and “some” (23/46, <50%). Where participants expressed multiple perspectives, responses were coded under all relevant categories to reflect the complexity of AI integration in practice. This structured and multisource approach ensured the robustness, transparency, and trustworthiness of the findings.

### Trustworthiness

To ensure trustworthiness, the study systematically addressed the 4 key criteria of qualitative rigor: credibility, confirmability, dependability, and transferability. Credibility was strengthened through triangulation of data sources—semistructured interviews, focus groups, and AI-generated teaching artifacts—which provided multiple perspectives on the phenomenon. In addition, member checking was conducted by inviting participants to review selected transcripts and thematic summaries to validate interpretations.

The study used a hybrid coding approach, integrating both deductive and inductive strategies. A preliminary codebook based on the I-PACE model guided the initial round of coding, allowing the researchers to identify theoretically grounded patterns. Simultaneously, the analysis remained open to emergent inductive themes not captured by the model, thereby ensuring that the coding process was both conceptually informed and data-driven.

Confirmability was supported through independent coding by 2 researchers on a subset of transcripts (20/60, 30%), followed by peer debriefing and consensus-building sessions to refine code definitions and resolve discrepancies. While no formal interrater reliability statistic (eg, Cohen Kappa) was calculated, a shared audit trail was maintained to document analytic decisions, code evolution, and theme development. Final coding was conducted collaboratively using NVivo software.

Dependability was reinforced by clearly outlining data collection and analysis procedures, adhering to Braun and Clarke’s 6-phase thematic analysis protocol, and using NVivo for systematic coding and data management. Transferability was supported through purposive sampling for maximum variation across discipline, institutional context, and AI use frequency, along with thick descriptions of participants’ backgrounds and contextual settings. Linguistic and cultural nuances were preserved through bilingual coding and backward translation, ensuring integrity across languages. Collectively, these procedures establish a rigorous foundation for the trustworthiness of the study’s findings.

### Ethical Considerations

The study was conducted following approval (approval number Med. Dec. 2024/29) from the institutional review board at An Najah National University. All participants provided informed consent, receiving clear explanations of the study’s purpose, voluntary participation, and confidentiality. Consent was indicated by participation, and participants were reminded of their right to withdraw at any time, ensuring ethical compliance and autonomy.

### Researchers' Background and Positionality

This study was conducted by an interdisciplinary research team composed of scholars from diverse academic backgrounds and institutions across different countries. The team includes experts in medical education, English language studies, educational technology, and qualitative research methodology, with shared experience in the integration of AI tools in teaching, learning, and research. Members of the team are affiliated with universities in the Middle East and North Africa region, providing a transnational perspective on the evolving role of AI in higher education. The team’s varied disciplinary expertise and familiarity with a wide range of learner populations—including undergraduate students, clinical educators, and faculty across Science, Technology, Engineering, and Mathematics (STEM) and humanities disciplines—helped ensure a nuanced and contextually grounded interpretation of the data. This interdisciplinary orientation aligns with the study’s broader aim to explore AI dependency as a multidimensional and cross-sectoral phenomenon.

## Results

### Overview

To contextualize the qualitative findings, Table 1 presents the demographic characteristics of the 46 participants who took part in the study, including faculty from diverse medical and health sciences disciplines such as nursing, pharmacy, optometry, medicine, and dental sciences. Participants varied in gender, age, teaching experience, frequency of AI use, and level of AI familiarity, ensuring a wide range of perspectives on the phenomenon of AI dependency in academic practice.

**Table 1.** Demographic information about the participants in the semistructured interviews and focus group sessions.

Variable	Frequency (%)
Sex	
Male	20 (43.5)
Female	26 (56.5)
Age (years)	
25‐35	5 (10.9)
36‐45	15 (32.6)
46‐55	18 (39.1)
56+	8 (17.4)
Frequency of gen AI^a^ use	
Daily	21 (45.7)
Weekly	14 (30.4)
Monthly	7 (15.2)
Occasionally	4 (8.7)
Teaching experience	
1‐5 years	16 (34.8)
6‐10 years	6 (13.0)
11‐15 years	15 (32.6)
16+ years	9 (19.6)
AI familiarity level	
Low	19 (41.3)
Moderate	16 (34.8)
High	11 (23.9)
Medical science discipline	
Nursing	8 (17.4)
Pharmacy	8 (17.4)
Optometry	9 (19.6)
Doctor of Medicine	7 (15.2)
Medical Laboratory Sciences	6 (13.0)
Doctor of Dental	8 (17.4)

a AI: artificial intelligence.

Building on this diverse dataset, the findings are structured and interpreted through the lens of the I-PACE model. This framework enabled a systematic mapping of themes across 4 interrelated components: institutional and individual pressures were aligned with person variables; anxiety and motivation reflected the affect dimension; cognitive offloading and creativity suppression were linked to cognition; and habitual overuse of AI tools corresponded to execution functions. Together, these findings illustrate how psychological, cognitive, and behavioral mechanisms interact to shape educators’ dependency on AI technologies in higher education.

Research question #1*:* What key factors contribute to AI dependency among educators in higher education?

The first research question aimed to explore the factors influencing AI dependency among educators. The analysis revealed that AI dependency is shaped by institutional, psychological, cognitive, technological, and individual factors. Table 2 presents the coding book for the first research question.

**Table 2.** Coding book for the reasons for AI dependency research question.

Theme and subtheme	Quotation
Institutional factors	
Heavy workload and time constraints	*Without AI, I would spend hours grading assignments and drafting course materials. Now, I can do it in minutes*. [E5]
Institutional expectation of using AI^a^	*Our university expects us to use AI, but there’s little guidance on how to do it effectively*. [E21]
Lack of institutional guidance	*There are no clear policies on how much AI use is appropriate, so I just use it however I think best*. [E8]
Psychological factors	
Anxiety and performance pressure	*Everyone around me is using AI, and I don’t want to be left behind.* [E2]
Perfectionism and fear of errors	*I feel like I’ve lost my ability to draft a paper from scratch. I always turn to AI first*. [E6]
Cognitive factors	
Cognitive offloading	*I used to brainstorm lesson plans myself. Now, I ask AI, and it gives me something within seconds*. [E15]
Loss of pedagogical creativity	*I used to create my own case studies and interactive activities. Now, I just modify what AI generates*. [E17]
Technological factors	
Ease of access and automation	*Why spend hours writing something when AI can give me a great draft in seconds?* [E21]
Lack of AI literacy	*I know AI isn’t perfect, but I don’t always know how to verify the accuracy of what it generates*. [E18]
Feeling powerful and capable	*With AI, I can complete tasks that would have taken me hours in just a few minutes*. [E3]
Individual factors	
Academic self-efficacy	*AI helps me refine my ideas, making my work more professional and well-structured*. [E6]
Academic reputation	*I rely on AI in writing research papers due to my reputation and publication pressure*. [E9]
High performance expectations	*There’s a lot of pressure to produce high-quality work, so I use AI to meet expectations*. [E11]
Low academic confidence	*I don’t always trust my writing skills, so AI gives me the reassurance I need to finalize my work*. [E4]
Lack of scientific research engagement	*I rarely engage in deep scientific research anymore because AI provides quick summaries*. [E13]

a AI: artificial intelligence.

### Institutional Factors

#### Heavy Workload and Time Constraints

Many participants viewed AI as essential for managing academic workloads, highlighting its role in reducing time spent on repetitive tasks like grading, report writing, and content creation.

*Without AI, I would spend hours grading assignments and drafting course materials. Now, I can do it in minutes, which helps me keep up with my workload.*

[E5]

Participants noted that institutional pressure to publish and show teaching effectiveness drives greater AI adoption. However, some—especially in the social sciences—found that unfamiliarity with AI increased their workload and added stress.

*It’s a double-edged sword—AI helps me finish tasks faster, but I sometimes feel I need more time to understand it, which increases the time I need to finish writing the tasks.*

[E19]

#### Lack of Institutional Guidance

A recurring concern was the absence of institutional policies, support, or structured training for integrating AI responsibly. This lack of formal guidance often left participants unsure of how to use AI tools effectively and ethically.

*There are no clear policies on how much AI use is appropriate, so I just use it however I think best.*

[E8]

A few also expressed concern that future policy changes might disrupt their current dependency on AI.

*I depend on AI now, but if my university decides to impose restrictions, I might struggle to adjust back to traditional methods.*

[E5]

### Psychological Factors

#### Anxiety and Performance Pressure

Several participants pointed out that AI helps them manage anxiety related to teaching and research expectations. Some also reported feeling pressured to stay updated with AI advancements and integrating AI tools in order to be adequately tech-savvy.

*Everyone around me is using AI, and I don’t want to be left behind. I feel like I need to use it to stay relevant.*

[E2]

Nevertheless, a few participants argued that AI dependency sometimes increases stress, particularly when the accuracy of AI is uncertain.

*I depend on AI, but at times, I worry that I might be using incorrect or biased information without realizing it.*

[E9]

#### Perfectionism and Fear of Errors

Some participants described themselves as perfectionists, adding that AI helps them ensure accuracy in research, lesson planning, and assessment design. In addition, AI increases their confidence as it helps them refine content and eliminate errors.

*I use AI to check everything. I don’t want to submit anything that isn’t polished and error-free*.

Conversely, a few expressed concern over the way AI dependency negatively impacts their academic and scholarly confidence.

*I feel like I’ve lost my ability to draft a paper from scratch. I always turn to AI first.*

[E 6]

### Cognitive Factors

#### Cognitive Offloading

Most participants acknowledged that AI has become their default tool for information retrieval and content creation, reducing the effort required for critical thinking and problem-solving.

*I used to brainstorm lesson plans myself. Now, I ask AI, and it gives me something within seconds.*

[E15]

However, some educators worried that relying on AI too frequently might weaken their cognitive engagement with their work.

*I wonder if I’m losing my ability to think critically because AI does the thinking for me.*

[E 10]

#### Loss of Pedagogical Creativity

A few pointed out that AI-generated content makes teaching more convenient but limits their creativity in designing course materials.

*I used to create my own case studies and interactive activities. Now, I just modify what AI generates.*

[E17]

On the other hand, some educators mentioned that AI enhances their creativity by providing new perspectives and ideas they would not have considered otherwise.

*AI gives me different angles to approach a topic, which actually improves my lesson planning.*

[E22]

### Technological Factors

#### Ease of Access and Automation

Many participants indicated that AI tools are readily available and easy to use, making them convenient for performing academic tasks. According to these participants, AI helps them save time, generate ideas, and organize content efficiently.

*Why spend hours writing something when AI can give me a great draft in seconds?*

[E21]

However, a few pointed out that the ease of access makes it tempting to rely on AI for everything, leading to unintentional dependency.

*The more I use AI, the harder it becomes to work without it. It’s like having a calculator for everything.*

[E20]

#### Lack of AI Literacy

Some participants admitted that they lack a deep understanding of AI’s inner workings**,** leading them to unquestioningly trust its outputs.

*I know AI isn’t perfect, but I don’t always know how to verify the accuracy of what it generates.*

[E18]

A few educators expressed concern that inadequate AI literacy might make them overly dependent on AI-generated insights.

*I sometimes use AI without thinking critically because I assume it knows better than I do.*

[E1]

#### Feeling Powerful and Capable

Some educators pointed out that AI empowers them and allows them to produce high-quality content more efficiently.

*With AI, I can complete tasks that would have taken me hours in just a few minutes. It makes me feel more capable.*

[E3]

Moreover, a few were concerned that this sense of control is misleading, as they might be overestimating AI’s reliability.

*AI makes me feel smarter, but I sometimes wonder if I’m just relying on it too much without questioning its accuracy.*

[E 3]

#### Individual Factors

Based on the coding book (Table 2), individual factors include academic reputation, high performance expectation, low academic self-efficacy, and lack of scientific research engagement.

### Academic Reputation

Most participants reported that academic reputation is a key driver of their reliance on AI, particularly for scientific research. One prominent researcher shared that, because of his demanding teaching and research schedule, he relies on AI to assist with writing.

### Academic Self-Efficacy

Some participants mentioned that using AI enhances their confidence in their academic abilities.

*AI helps me refine my ideas, making my work more professional and well-structured.*

[E6]

Other participants expressed concerns that relying on AI might affect their academic reputation, as AI-generated content could be perceived as lacking originality.

*I worry that using AI too much might make others question the authenticity of my work.*

[E7]

#### Low Academic Confidence

A few participants admitted that AI serves as a tool for procrastination, allowing them to delay tasks while still producing quick results when needed.

*“I put off writing papers because I know AI can help me generate content at the last minute.”*

On the other hand, some educators maintained that AI use compensates for their low academic confidence, making them feel more capable in completing their work.

*“I don’t always trust my writing skills, so AI gives me the reassurance I need to finalize my work.”*

Research question #2: How does AI dependency impact educators’ teaching autonomy, decision-making, and professional identity?

Findings from the second research question revealed that AI dependency has complex and varied consequences for educators. While participants acknowledged AI’s efficiency and convenience, they also raised concerns about its negative effects. More than 30 subthemes emerged, categorized into 6 main themes: skills atrophy, pedagogical erosion, motivation decline, ethical and integrity risk, social fragmentation, and creativity suppression. Table 3 details these subthemes, which are outlined in the following sections.

**Table 3.** Consequences of AI dependency.

Main theme	Subtheme
Skills atrophy	Loss of critical thinking Weakened problem-solving abilities Diminished writing and research skills Reduced analytical skills Loss of decision-making abilities Reduced ability to synthesize independently Weakened judgment
Motivational decline	Over-reliance on AI^a^ for simple tasks Procrastination Reduced motivation Increased laziness Reduced individual initiatives
Pedagogical erosion	Erosion of pedagogical autonomy Reduced active engagement with students Weakened academic mentorship Reduced self-confidence Perceived professional inadequacy
Ethical and integrity risks	Increased plagiarism rate Increased copyright infringement Increased academic misconduct Diminished human responsibility Increased misleading information
Social fragmentation	Affects students’ emotional state negatively Diminished social development Reduced human interaction Decreased collaboration among humans
Creativity suppression	Homogenization of knowledge production Restrict creativity Restrict information seeking

a AI: artificial intelligence.

### Skills Atrophy

In this study, skills atrophy refers to the gradual decline of previously acquired skills and competencies due to lack of use or engagement. Related subthemes include weakened thinking skills, reduced problem-solving abilities, and diminished analytical synthesis.

All participants in the interviews and a few of the participants in the focus group discussions reported a decline in their skills in writing scientific research due to their frequent use of AI in scientific research. In addition, most participants reported that frequent reliance on AI has reduced their engagement in deep critical thinking.

*I used to spend time reading multiple sources and forming my own analysis. Now, I just ask AI to summarize everything for me, and I take that as my starting point.*

[EFG18]

Several participants in the interviews admitted that instead of independently evaluating and synthesizing information, they now trust AI-generated summaries and responses.

*I don’t challenge myself as much anymore. If AI gives me a structured answer, I don’t always question it, I just go with it.*

[EFG26]

In addition, several participants stated that using AI applications negatively affects their decision-making abilities, making them more reliant on automated solutions.

*When I come across a difficult question in my field, my first instinct is to ask AI instead of thinking through possible solutions myself.*

[EFG36]

A few participants pointed out that while AI speeds up the problem-solving process, it sometimes limits their ability to engage with complex academic challenges.

*AI gives great answers quickly, but I worry that I’m losing my ability to navigate difficult academic discussions without its help.*

[EFG13]

Most participants reported that AI dependence has weakened their academic writing and research skills. Some found it difficult to start writing without AI-generated drafts after extended use, while others rarely conduct extensive literature reviews themselves.

*I used to write everything from scratch, but now I start with an AI-generated outline. I wonder if I’m losing my ability to structure my thoughts clearly on my own.*

[EFG25]

A few participants mentioned that AI-generated summaries are convenient but may lead to a superficial understanding of academic topics.

*Instead of reading full papers, I rely on AI to summarize them. I get the key points, but I feel like I’m missing the depth of the discussion.*

[E17]

### Motivational Decline

In the context of this study, motivation decline refers to the gradual reduction in an individual’s drive, enthusiasm, or willingness to engage in various academic activities. Subthemes include procrastination, weaker intrinsic motivation, reduced individual initiatives, and overreliance on AI.

Several participants mentioned that AI has encouraged procrastination, as they know they can complete tasks quickly with AI assistance.

*Before AI, I would start preparing my lectures well in advance. Now, I just rely on AI to generate material the night before.*

[EFG28]

A few educators pointed out that while AI helps them meet deadlines, it sometimes leads to rushed and poor-quality work.

*I meet my deadlines, but sometimes I feel like my work isn’t as thoughtful as it used to be because I rely on AI to speed things up.*

[E15]

A few educators mentioned that AI has made them more efficient but acknowledged that it can be tempting to take shortcuts.

*I’m more productive with AI, but I also recognize that I use it to avoid thinking through certain problems myself.*

[EFG13]

### Pedagogical Erosion

In this study, pedagogical erosion refers to the gradual decline in the effectiveness, relevance, and innovation of teaching practices over time. Subthemes categorized under this theme include perceived professional inadequacy, erosion of pedagogy autonomy, reduced engagement with students, weakened academic mentorship, and reduced self-confidence.

Some participants pointed out that AI efficiently helps them improve the quality of their teaching material.

*“AI gives me a strong starting point for my lessons. I still personalize them, but it definitely saves me time.”*

According to a few participants, interactions with students became less personal since integrating AI into their workflow.

*I used to spend more time giving individualized feedback. Now, I use AI-generated comments, and I feel like I’m not engaging with my students as much.*

[E11]

On the other hand, some educators argued that AI allows them to focus on more meaningful discussions instead of wasting time on repetitive tasks.

*AI handles the routine work, so I can dedicate more time to having deeper discussions with my students.*

[E20]

Many participants admitted that AI has made them doubt their own expertise, as they often feel that AI-generated content is superior to their own in structure and insight.

*Sometimes I feel like AI writes better than I do. It’s unsettling to think that I might not be as good as I used to be.*

[EFG35]

Similarly, some mentioned that they feel they must use AI particularly when completing research or complex writing tasks.

“*If AI was suddenly unavailable, I don’t know how I’d manage my workload. I’ve started to rely on it too much*.”

However, a few pointed out that AI boosts their confidence by helping them refine their work.

AI isn’t replacing my skills—it’s just giving me an extra layer of support to make my work better.

[EFG14]

A few educators stressed that AI can and should be used wisely without hindering professional competence.

*AI is a tool, not a replacement. As long as we use it strategically, we can avoid becoming too dependent.*

[E16]

### Ethical and Integrity Risks

Ethical and integrity risks in this study refer to the challenges that arise when AI influences academic practices. Subthemes that emerged under this category include increased plagiarism rate and copyright infringement, increased academic misconduct, decreased human responsibility, and increased misleading information.

Some participants expressed concerns that AI facilitates plagiarism and reduces students’ accountability for their work.

*It’s so easy for students to copy-paste AI-generated responses without truly engaging with the material.*

[E7]

Others pointed out that AI tools blur the lines of originality and authorship, making it difficult to differentiate between human and machine-generated work.

*I worry that students are submitting AI-generated essays without understanding the concepts themselves.*

[E12]

A few highlighted the fact that AI-generated content sometimes lacks accountability, as it can produce misleading or biased information.

*AI sometimes generates convincing but factually incorrect statements, and students don’t always verify them.*

[EFG21]

On the other hand, some faculty members believe AI can be ethically leveraged if students are taught responsible AI use.

*We should integrate AI literacy into our curriculum so students learn how to use these tools without compromising integrity.*

[E26]

### Social Fragmentation

Social fragmentation in this research refers to the weakening of human connections and interactions due to AIED. Subthemes include negatively impacting students’ emotional state, diminishing social development, reducing human interaction, and decreasing collaboration among humans.

Many participants noted that students are becoming more isolated as AI tools replace traditional peer interactions in collaborative assignments.

*Group discussions are not as engaging anymore because students rely on AI instead of exchanging ideas with their peers.*

[E9]

However, some suggested that AI could be used to enhance social learning if implemented thoughtfully.

*If we integrate AI strategically—such as using it to facilitate discussions rather than replace them—it can actually strengthen collaboration.*

[E30]

### Creativity Suppression

Creativity suppression refers to the risk that AI may homogenize knowledge production, restrict creativity, and limit students’ ability to seek information. Subthemes include homogenizing knowledge production, restricting creativity, and limiting search for information from diverse sources.

Many participants reported that AI-generated content tends to produce generic, standardized responses, leading to a decline in original thought.

*Students’ assignments are starting to look the same because they rely on AI-generated structures.*

[EFG15]

Some educators emphasized that AI discourages thorough research, as students may settle for AI-generated summaries instead of exploring diverse sources.

*Before AI, students would read multiple papers. Now, they just use AI to summarize, and I feel like they’re missing out on critical engagement.*

[E14]

Research question # 3: What strategies can help educators balance AI use while maintaining pedagogical creativity and instructional effectiveness?

The third research question explored strategies educators use to balance AI integration with traditional teaching while maintaining effectiveness and autonomy. Findings show that most view AI as a valuable tool but use strategies to prevent overreliance and preserve human-centered teaching, with some embracing its efficiency and others expressing concerns about its effects on critical thinking, creativity, and student-teacher interactions. Key themes are summarized below.

### Establishing Clear Boundaries for AI Use: Selective Use of AI for Instructional Tasks

Most participants said they use AI selectively, viewing it as a support tool rather than a replacement for their instructional role. They emphasized that core teaching activities—mentoring, leading discussions, and assessing student progress—should remain human-led.

*AI helps with structuring lesson plans and summarizing content, but I make sure that my role as an educator remains central in guiding discussions and engaging with students.*

[E4]

Some participants pointed out that AI is particularly useful for administrative tasks and content organization but should not dictate teaching methods.

*I let AI handle routine tasks like scheduling and summarizing, but when it comes to interactive teaching, I rely on my own expertise and instincts.*

[EFG33]

A few participants expressed concerns that without defined limits, educators might unconsciously begin to depend too much on AI-generated content.

*I initially used AI just for support, but over time, I found myself relying on it more than I intended. Now, I consciously limit my AI use to avoid dependency.*

[EFG28]

### Encouraging Critical Engagement With AI: Verifying AI-Generated Content

Many participants stated that they actively verify and refine AI-generated outputs before using them in teaching, noting that AI can produce misleading, biased, or overly simplified content. This requires educators to serve as content curators rather than passive users.

*I never use AI-generated content without reviewing it first. It can be a great starting point, but I always fact-check and refine the materials to align with my course objectives.*

[EFG31]

Some educators pointed out that they encourage students to critically analyze AI-generated responses rather than accepting them at face value.

*I tell my students that AI is a tool, not an authority. They need to critique what it produces, question its assumptions, and think beyond the outputs.*

[EFG32]

On the other hand, a few participants expressed concern that not all educators take the time to evaluate AI content carefully, which could lead to inaccuracies in teaching.

*One of my worries is that some educators might blindly trust AI-generated materials, which could introduce errors or outdated information into their lessons.*

[EFG17]

### Balancing AI With Human-Centered Teaching

#### Hybrid Intelligence

Most participants stressed that hybrid intelligence—integrating human and machine intelligence—is vital to prevent overreliance on AI. They defined it as critically assessing AI outputs, promoting deeper reflection and independent analysis rather than passive acceptance.

#### Prioritizing Interactive and Discussion-Based Learning

Most participants reported intentionally designing courses to prioritize live discussions, debates, and student-driven learning to counterbalance AI-generated content, emphasizing that human interaction is essential for deep understanding and critical thinking.

*AI is great for providing structured information, but meaningful learning happens when students engage in discussions, challenge ideas, and interact with their peers and instructors.*

[E15]

Some participants mentioned that they use AI to supplement discussions by generating diverse perspectives, but they ensure that students analyze and debate those perspectives rather than passively accepting them.

*I sometimes use AI to provide multiple viewpoints on a topic, but I make sure my students critically evaluate and compare them rather than just absorbing the information.*

[EFG25]

However, a few participants pointed out that some educators struggle to balance AI integration with traditional teaching, leading to a more passive learning environment.

*I've seen cases where AI-generated lectures replace interactive teaching, and I worry that students might become passive learners rather than active thinkers.*

[EFG32]

### Maintaining Pedagogical Creativity: Using AI as a Spark for Innovation, Not a Substitute for Creativity

Many participants reported that they leverage AI to generate new ideas for lesson planning but ensure that their personal creativity remains the driving force. They view AI as a brainstorming partner rather than a content creator.

*I use AI to generate multiple lesson ideas, but I always customize them to fit my teaching style and my students' needs.*

[E3]

Some educators pointed out that AI helps them explore innovative approaches to teaching but emphasized that human creativity is irreplaceable.

*AI can suggest engaging activities, but it’s my job to refine them and add the human element that makes learning meaningful.*

[E22]

On the other hand, a few participants expressed concerns that excessive AI use might lead to a decline in originality if educators become overly reliant on AI-generated content.

*I fear that if we depend too much on AI, we might lose the uniqueness of our teaching styles. That’s why I try to balance AI use with my own creative input.*

[EFG26]

### Fostering Student AI Literacy and Ethical Use: Teaching Students to Use AI Responsibly

Some participants expressed that they incorporate AI literacy into their courses to help students use AI effectively while avoiding over-reliance. They believe that educators should guide students in understanding AI’s strengths and limitations.

*I teach my students how to use AI as a research tool but also emphasize that they must engage critically with the results rather than just accepting AI-generated answers.*

[EFG32]

Most participants reported that they actively discourage students from using AI for academic shortcuts, instead encouraging them to use AI as a means of enhancing learning.

*AI can help students brainstorm ideas, but they need to put in the effort to analyze and expand on those ideas instead of just submitting AI-generated content.*

[EFG34]

However, a few participants pointed out that some students misuse AI for assignments, and educators must establish clear guidelines on ethical AI use.

*We need to teach students that AI is a tool to assist learning, not a way to bypass intellectual effort.*

[EFG11]

### Continuous Professional Development and Peer Collaboration: Engaging in AI Training and Professional Learning Communities

Several participants mentioned that they actively seek professional development opportunities to improve their AI literacy and refine their AI integration strategies. They believe that educators must continuously learn to ensure that AI is used responsibly and effectively.

*I regularly attend AI workshops to stay updated on best practices. The more I understand AI, the better I can integrate it into my teaching without becoming dependent on it.*

[E5]

Some educators pointed out that they collaborate with colleagues to share AI integration strategies, discuss ethical concerns, and develop guidelines for responsible AI use.

*We have faculty discussions on AI use where we exchange ideas on how to integrate AI without compromising teaching quality. These discussions help us find the right balance.*

[EFG36]

On the other hand, a few participants expressed that some institutions lack adequate AI training programs, leaving educators to navigate AI integration on their own.

*I wish there were more structured AI training for educators. Right now, we mostly figure it out through trial and error.*

[EFG35]

## Discussion

### Overview

The recent study examines the drivers and consequences of AI dependency among medical and health sciences faculty in Palestinian higher education, offering critical insights into over-reliance on generative AI and strategies for sustainable integration. This study contributes to the growing body of literature on digital transformation in medical education by identifying multidimensional factors that influence educators’ reliance on AI tools and their implications for pedagogical integrity and professional agency.

### Principal Findings

Institutional pressures—particularly workload intensity and a lack of clear AI governance—were identified as primary catalysts for increased AI reliance. These findings mirror previous studies [4,10] highlighting how time-saving AI applications appeal to overburdened faculty. Participants described using AI to manage grading, feedback, and administrative documentation, often without structured support or guidance. The absence of institutional frameworks creates ambiguity, allowing overuse and reinforcing dependency—a concern consistent with the findings of [7].

Psychological and individual factors also emerged as significant influences. Echoing previous research [8,9,32], educators reported that anxiety, performance pressure, and perfectionism motivated AI use as a coping mechanism. High expectations for output quality and academic reputation intensified AI reliance, particularly in competitive or high-stakes disciplines. While some participants viewed AI as a confidence booster, others reported reduced academic self-efficacy and increased procrastination, reflecting problematic patterns of technology use.

At the cognitive level, many educators admitted to cognitive offloading, allowing AI to complete tasks once handled through personal reflection or pedagogical creativity. These practices align with concerns from [33,34] about diminished analytical and innovative capacity when AI is used uncritically. Participants noted that AI-generated lesson plans increased efficiency but limited deep thinking and originality, a paradox especially concerning in health education where critical reasoning and ethical judgment are essential.

Technological factors, especially ease of access and automation, facilitated habitual AI use, often unconsciously. Similar to findings on digital tool overuse [25], educators recognized both the benefits and risks of AI ubiquity. Lack of AI literacy compounded the issue: while participants used GenAI tools regularly, many admitted limited understanding of how algorithms operate or how to evaluate their outputs critically [17]. This blind trust not only undermines informed decision-making but also amplifies the risk of propagating misinformation or algorithmic bias in academic work.

### Consequences of AI Dependency

This study identified 6 major consequences of AI overreliance: skills atrophy, motivational decline, pedagogical erosion, ethical risks, social fragmentation, and creativity suppression. Faculty reported declining engagement in original writing, idea generation, and academic rigor—findings echoed by [8,34,35]. In some cases, AI was used as a substitute for personal initiative, weakening academic identity and self-reliance.

Social fragmentation was also discussed as educators expressed concern about diminished student-teacher interaction and reduced peer collaboration. This is consistent with [36] noting how digital tools can isolate learners. Our findings suggest AI can support collaborative learning by matching students for group work and distributing roles equitably, potentially countering its fragmenting effects.

Participants frequently cited ethical risks such as plagiarism, misinformation, and authorship concerns, expressing uncertainty about acceptable boundaries for AI use in assessment and publication. These concerns align with calls for regular AI audits [34], ensuring educational technologies are ethically vetted and contextually appropriate.

### Comparison to Prior Work

The findings reinforce and expand on theoretical models such as the I-PACE framework [12], which links personality traits, affective responses, and cognitive control to problematic technology use. This study shows that in Palestinian higher education, AI dependency is a systemic issue shaped by resource constraints and institutional pressures, not just individual behavior. It highlights the interplay between personal motivations and structural limitations in AI use.

### Limitations and Future Research

This study’s findings are limited by its focus on Palestinian higher education, which may affect transferability to other contexts. Variations in resources, policies, and academic cultures could lead to different experiences with AI dependency. Future research should explore these issues in diverse settings to broaden understanding.

Second, the use of self-reported data from 46 faculty members may introduce social desirability bias, with participants potentially misrepresenting their AI use. Conducting and translating interviews from Arabic to English may also have affected the preservation of linguistic and cultural nuances. Future studies should consider bilingual coding teams and cultural consultants for greater interpretive accuracy.

Third, this study focused solely on educators’ perspectives, excluding student voices. Future research should examine AI dependency among students to understand its effects on motivation, learning outcomes, and academic integrity.

Furthermore, while this study examined AI dependency broadly, it did not address disciplinary differences. Faculty in humanities, social sciences, and STEM may experience unique challenges and opportunities with AI. Future research should compare disciplines to uncover domain-specific dynamics of AI dependency.

Finally, this study provides a snapshot of current practices but does not assess the long-term effects of AI dependency on faculty roles, creativity, or knowledge production. Longitudinal and mixed methods research is needed to track changes over time and evaluate the impact of institutional interventions on pedagogical autonomy and professional integrity.

### Theoretical and Practical Implications

This study contributes to theoretical discourse on technology adoption in higher education by framing AI dependency as a multidimensional construct shaped by institutional, psychological, cognitive, and technological factors. Building on the I-PACE model, the findings demonstrate how overreliance on generative AI aligns with patterns of problematic technology use, particularly among faculty navigating high academic demands and limited institutional support. The study reinforces and extends the I-PACE framework by showing how its four components—Person, Affect, Cognition, and Execution—interact dynamically within educational environments to shape AI dependency.

Under the Person component, both individual (eg, academic self-efficacy, perfectionism, low confidence) and institutional drivers (eg, workload pressures, unclear policies) were identified as predisposing factors for AI overuse. These findings extend I-PACE by highlighting that external academic structures—not just personal traits—can significantly shape technology dependency. The Affect domain was reflected in emotional responses such as anxiety, fear of inadequacy, and pressure to meet performance expectations. Rather than being purely internal, these emotions were often shaped by systemic academic stressors, suggesting that affective triggers in the I-PACE model must be understood within broader socio-institutional contexts.

In the Cognition domain, participants frequently described cognitive offloading and declining engagement in creative and reflective tasks. Notably, this study introduces “creativity suppression” as a cognitive outcome not emphasized in the original model, pointing to the need for an update to account for AI’s influence on ideation and pedagogical originality. The Execution component was evident in behavioral outcomes such as diminished teaching autonomy, motivational decline, and habitual reliance on AI. These confirm I-PACE’s focus on maladaptive behavior patterns but also reveal participants’ active strategies—like hybrid intelligence and critical engagement—to retain pedagogical agency.

Overall, the study affirms the I-PACE model’s value while proposing a broader interpretation that incorporates institutional, technological, and cultural factors specific to under-resourced higher education systems. By doing so, it deepens our understanding of how AI transforms academic identity, decision-making, and autonomy in the evolving landscape of digital education.

On a practical level, the study offers several actionable recommendations:

### Faculty Development

Higher education institutions should invest in personalized professional development to enhance AI literacy, ethical awareness, and pedagogical resilience. Training should include modules on AI ethics, data privacy, and algorithmic bias, tailored to each discipline’s needs. Institutions can also use AI to personalize learning pathways for educators based on their backgrounds and evolving instructional needs.

### Institutional Policy Design

Institutions must establish clear and adaptive AI governance frameworks to guide responsible AI use among faculty and students. These frameworks should define acceptable use policies, clarify the boundaries of AI-generated content, and promote transparency in AI-supported academic work. To uphold ethical standards—especially in high-stakes fields like medical education—regular audits of AI tools should be implemented to assess potential biases, misinformation, and integrity risks. Moreover, developing such policies should be a collaborative effort involving faculty members, administrators, IT professionals, and ethicists to ensure both institutional trust and community-wide compliance.

### Student–Teacher Interaction in AI Contexts

To reduce risks of pedagogical erosion and social fragmentation, institutions should set guidelines that preserve student–teacher engagement in AI-rich environments. Human–AI collaboration, reflective use of AI, and AI-facilitated group work can enhance accountability and peer interaction. Prioritizing feedback, mentorship, and dialogic teaching ensures AI remains a supplement, supporting balanced integration and safeguarding academic integrity.

### Conclusion

This study provides a comprehensive exploration of AI dependency among educators, highlighting the institutional, psychological, and cognitive factors that contribute to reliance on AI in academic practices. While AI offers significant benefits in terms of efficiency, knowledge management, and academic productivity, unchecked dependence can pose risks to pedagogical autonomy, critical thinking, and professional identity. The findings emphasize the importance of hybrid intelligence as a strategy to balance AI use, ensuring that technology enhances rather than replaces human intellectual engagement. The study’s contributions to the theoretical discourse on AI adoption extend existing models by integrating the concept of AI dependency within a broader framework of professional agency and academic integrity. From a practical standpoint, these findings underscore the need for institutional policies, faculty training, and AI literacy initiatives that foster responsible AI use. Ultimately, this study advocates for a balanced approach where AI serves as a tool to augment human intelligence rather than replace it. Future research should continue to explore AI’s evolving role in higher education, examining its long-term impact on faculty development, student learning, and knowledge creation in an increasingly AI-driven academic landscape.

## Supplementary material

10.2196/74947 Multimedia Appendix 1 Semistructured protocol.

10.2196/74947 Multimedia Appendix 2 Focus group discussion guide.

## Abbreviations

AI: Artificial Intelligence
AIED: Artificial Intelligence in Education
Gen AI: Generative Artificial Intelligence
IPACE: Interaction of Person-Affect-Cognition-Execution
STEM: Science, Technology, Engineering, and Mathematics
