# Supplementary material for: Paradox of AI in Higher Education: Qualitative Inquiry Into AI Dependency Among Educators in Palestine
Source: JMIR Med Educ. 2025 Sep 15;11:e74947. doi: 10.2196/74947 (PMC12435755; doi:10.2196/74947)
Supplement: Multimedia Appendix 1 [file mededu-v11-e74947-s001.docx]

**Appendix A**

**Semi-Structured Protocol**

Introductory Note to Participants
*This interview is part of a study exploring the use of generative AI in higher education. We are interested in understanding your experiences and perspectives as an educator using AI in your teaching, research, or administrative work. There are no right or wrong answers. Feel free to share openly.*

1. Can you describe how you first started using AI tools (e.g., ChatGPT) in your academic work?
2. What factors initially motivated you to try or adopt AI tools in your teaching, research, or administration?
3. How would you describe your confidence or skills in using AI tools before you began using them regularly?
4. In what ways do your professional goals or academic expectations influence your use of AI?
5. Have any institutional expectations or pressures affected your decision to use or avoid AI tools?
6. How do you usually feel when you are using AI tools in your work—relieved, anxious, unsure, confident? Why?
7. Have there been situations where you felt pressure to use AI to meet deadlines or maintain quality?
8. Do you ever worry about making mistakes in your academic work? How, if at all, does AI help or worsen that feeling?
9. Can you describe any emotional challenges or stress you associate with using or depending on AI tools?
10. What types of academic tasks do you typically delegate to AI (e.g., drafting text, generating ideas, summarizing)?
11. Do you find yourself thinking differently when you use AI—for example, relying on it to start or finish your ideas?
12. Can you think of a time when using AI helped—or hindered—your creativity or critical thinking?
13. How do you decide when to use AI versus when to work without it?
14. How often do you find yourself using AI tools now compared to when you first started?
15. Have your teaching or academic habits changed in any noticeable ways since you began using AI?
16. Do you think your use of AI has affected your teaching autonomy or academic identity? In what ways?
17. Are there any risks or drawbacks you’ve personally experienced or noticed in your AI use?
18. What do you consider responsible or ethical use of AI in your role as an educator?
19. Have you developed any personal guidelines or boundaries to avoid over-reliance on AI?
20. What kinds of support (training, policies, peer dialogue) would help you use AI more effectively and responsibly?
21. What advice would you give to other educators about balancing AI use with human teaching practices?
