# Supplementary material for: Paradox of AI in Higher Education: Qualitative Inquiry Into AI Dependency Among Educators in Palestine
Source: JMIR Med Educ. 2025 Sep 15;11:e74947. doi: 10.2196/74947 (PMC12435755; doi:10.2196/74947)
Supplement: Multimedia Appendix 2 [file mededu-v11-e74947-s002.docx]

**Appendix B**

**Focus Group Discussion Guide**

**Facilitator Introduction:**
Thank you for participating in this focus group. We are exploring how generative AI tools are shaping your work as educators—especially in teaching, research, and academic decision-making. These prompts are designed to spark open discussion based on your own experiences. Please feel free to respond to each other, build on ideas, or share contrasting views.

***Prompts***

1. Institutional workload, expectations, or the lack of policy guidance has shaped your use of AI in teaching, research, or administration—what do you think?
2. Based on your experience, how does your confidence, academic reputation, or professional background influence the way you use or depend on AI tools?
3. How have institutional culture and peer practices shaped your attitudes or behaviors toward AI use in your daily academic work?
4. Some educators experience anxiety, fear of making mistakes, or pressure to meet high standards—how do such emotional factors influence your use of AI?
5. Using AI can reduce stress for some but create uncertainty for others. Based on your experience, how has AI influenced your emotional state in academic work?
6. Perfectionism and performance pressure are common in higher education. How might these factors encourage increased reliance on AI tools?
7. Some educators find that AI helps them think more creatively, while others feel it limits their originality. How has AI influenced your own creativity and critical thinking?
8. Relying on AI for content generation or summaries can change how we approach problem-solving. In your experience, how has your cognitive engagement changed since using AI?
9. Can you reflect on whether you now use AI as a starting point for thinking, or if it’s replaced some of your original idea development?
10. Since you first began using AI, how has your frequency or pattern of use changed over time?
11. In what ways has your use of AI affected your teaching practices, assessment design, or decision-making routines?
12. Some educators feel that AI impacts their autonomy or professional identity. What changes have you personally noticed in your role or sense of agency since using AI?
13. To avoid becoming overly dependent on AI, some educators set personal limits or boundaries. Have you adopted any strategies to ensure a balanced use of AI?
14. From your perspective, what does responsible or ethical use of AI in education look like?
15. What institutional support—such as training, policy clarity, or peer collaboration—would help you use AI more effectively while preserving your autonomy and creativity?
16. How can educators promote a human-centered approach to AI use that still supports innovation and efficiency in the classroom?
17. Reflecting on everything we discussed, how do you see AI reshaping your identity or responsibilities as an educator in the future?
